# Technical efficiency of women’s health prevention programs in Bucaramanga, Colombia: a four-stage analysis

**DOI:** 10.1186/s12913-016-1837-0

**Published:** 2016-10-13

**Authors:** Myriam Ruiz-Rodriguez, Laura A. Rodriguez-Villamizar, Ileana Heredia-Pi

**Affiliations:** 1 Departamento de Salud Pública, Universidad Industrial de Santander, Carrera 32 No. 29-31 oficina 301, Bucaramanga, Colombia; 2 Instituto de Salud Pública de Mexico, Universidad 655, Santa María Ahuacatitlán, CP 62100 Cuernavaca, Morelos Mexico

**Keywords:** Technical efficiency, Primary health care, Data Envelopment Analysis, Prevention, Women, Colombia

## Abstract

**Background:**

Primary Health Care (PHC) is an efficient strategy to improve health outcomes in populations. Nevertheless, studies of technical efficiency in health care have focused on hospitals, with very little on primary health care centers. The objective of the present study was to use the *Data Envelopment Analysis* to estimate the technical efficiency of three women’s health promotion and disease prevention programs offered by primary care centers in Bucaramanga, Colombia.

**Methods:**

Efficiency was measured using a four-stage data envelopment analysis with a series of Tobit regressions to account for the effect of quality outcomes and context variables. Input/output information was collected from the institutions’ records, chart reviews and personal interviews. Information about contextual variables was obtained from databases from the primary health program in the municipality. A jackknife analysis was used to assess the robustness of the results.

**Results:**

The analysis was based on data from 21 public primary health care centers. The average efficiency scores, after adjusting for quality and context, were 92.4 %, 97.5 % and 86.2 % for the antenatal care (ANC), early detection of cervical cancer (EDCC) and family planning (FP) programs, respectively. On each program, 12 of the 21 (57.1 %) health centers were found to be technically efficient; having had the best-practice frontiers. Adjusting for context variables changed the scores and reference rankings of the three programs offered by the health centers.

**Conclusion:**

The performance of the women’s health prevention programs offered by the centers was found to be heterogeneous. Adjusting for context and health care quality variables had a significant effect on the technical efficiency scores and ranking. The results can serve as a guide to strengthen management and organizational and planning processes related to local primary care services operating within a market-based model such as the one in Colombia.

**Electronic supplementary material:**

The online version of this article (doi:10.1186/s12913-016-1837-0) contains supplementary material, which is available to authorized users.

## Background

Primary Health Care (PHC) is an efficient strategy to improve health outcomes for populations. Nevertheless, health systems in Latin America face different challenges in achieving health systems based on PHC [1, 2], including: insufficient human resources and training, lack of evaluations of the strategies’ outcomes, insufficient flexibilization and coordination of resources at management and operating levels and weaknesses in the public health system’s response capacity.

In Colombia, the General Social Security Health System (SGSSS, Spanish acronym) faces additional challenges derived from a decentralized, market-oriented health model based on managed competition and a managed care organizational structured [3–7]. These include a lack of efficiency, quality and effective access.

In Colombia, most of the PHC strategies are performed at the level of the Health Centers (HC), which are the heart of primary care at the first level of care in the public network [8]. Since HC are where most of the health promotion and disease prevention activities occur, they receive a large portion of the public budget allocated to population health at the municipal level and play an important role in offering services to a large proportion of the population throughout their lifecycle (45,5 % of the population of Bucaramanga is treated in public HC). Thus, it is important to evaluate the effectiveness, quality, equity and efficiency of the resources used for health care. The performance of HC is crucial to the functioning of the Colombian SGSSS and measuring their efficiency is important to prioritizing strategies that contribute to the sustainability of the SGSSS. Furthermore, PHC is known to play a key role in the financial sustainability, quality of care and health outcomes of health systems [9, 10].

Although HC are important to the population and to health systems in Colombia and worldwide, studies of technical efficiency have focused at the hospital level, with very few on PHC centers [4, 6, 7]. While it is important to identify and improve the technical efficiency of health institutions at all levels of care, it is more so for institutions such as HC, whose actions affect current and future generations [11–13].

Efficiency in the health sector increases the health gains that can result from optimizing the resources and processes involved in the production processes used by health service providers such as HC and hospitals. Technical efficiency refers to the physical relation between resources (capital and labour) and health outcome [14]. In others words, in the production of health care, health centers should act efficiently in terms of using their inputs to obtain maximum output. Technical efficiency is the ability of a firm to obtain maximal output for a given set of inputs [15, 16]. Measuring the efficiency of primary health service providers helps to identify, according to a particular standard, the institutions that best manage their resources and processes in order to attain optimal productions levels; and those that perform below capacity [17]. It also enables analyzing and selecting potential steps towards improvement. In addition, the results from this measurement can serve to strengthen management skills at the local level.

The *Data Envelopment Analysis* (DEA) is the strategy most often used to study the efficiency of health providers. This method is a non-parametric technique, which can be used to jointly study relationships among the resources used to provide health services and the total services offered over a period of time. It also estimates the efficiency level of a health center with respect to the other centers simultaneously included in the analysis. Therefore, the estimations of technical efficiency are considered to be relative rather than absolute, since they can vary when the set of centers changes, and a center that was efficient can become inefficient and vice versa [18] .

According to the DEA method, a production unit is technically inefficient when it can produce the same amount of outputs with a smaller amount of at least one input, or when it can use the same amount of inputs to generate more outputs [19].

Various studies of the technical efficiency of primary health care centers using the DEA method have been performed in the Americas [20–27]. These provide lessons regarding improvements that can be introduced at the centers studied. Nevertheless, as products they all evaluated office consultations and home visits by health teams as a whole, and none of these studies estimated the efficiency of women’s health care programs at HC.

The objective of the present study is to use the DEA analysis to measure the technical efficiency of 21 HC in the Bucaramanga metropolitan area, capital of Santander, Colombia, specifically in regard to three health promotion and disease prevention programs for women: Antenatal care (ANC), Family Planning (FP) and Early Detection of Cervical Cancer (EDCC). These HC operate as part of the Health Institute of Bucaramanga’s Social State Enterprise (ESE ISABU, Spanish acronym). The ESE ISABU is a public municipal health care provider that offers primary care services to the entire population enrolled in government-subsidized health insurance as well as the poor population that is unable to pay and has no health insurance.

It is crucial to evaluate the relative efficiency of HC so that their staff can quantitatively and graphically know to what degree they are achieving their objectives in comparison to peer centers, and to identify strengths and weaknesses to support strategies for improvement. To the best of the authors’ knowledge, this is the first study conducted to evaluate these primary care programs in Colombia.

Given the homogeneity of the programs studied, the findings from this investigation provide empirical evidence that may be of interest to Colombian health authorities in municipalities with characteristics similar to Bucaramanga. The findings also will contribute to proposing lines of work to improve the health care network in the context of new regulations for PHC through Resolution 429 issued by the government early in 2016, which is amending Law 1438 passed in 2011 and defines primary care as the health care strategy for Colombia.

## Methods

A cross-sectional, analytical, observational study was performed to estimate the technical efficiency of women’s health promotion and disease prevention programs offered by primary health care centers in Bucaramanga, the capital of Santander, Colombia. The investigation was performed between March 2013 and December 2014 in 21 of the 24 HC belonging to the ESE ISABU (Social State Enterprise at the primary care level). The women’s health care programs evaluated were low-risk Antenatal care (ANC), Family Planning (FP) and Early Detection of Cervical Cancer diagnostics program (EDCC). The health centers selected provide services to the population residing in 12 of the 17 districts in the Bucaramanga metropolitan area, which is characterized as poor, predominantly in the first and second socioeconomic strata and the majority is enrolled in government-subsidized health insurance.

### Study Area

Bucaramanga is a medium city with approximately 6000.000 inhabitants. It is nationally recognized for 97 % of its population having health coverage and 99 % of births delivered in institutions. The organization of the primary care services in the public sector in Bucaramanga adheres to national norms (the system’s organization is described in detail in the introduction of the article by Vargas et al.) [6]. It is decentralized and services are provided according to the SGSSS’s insurance model. The municipal health authority is the Bucaramanga Secretary of Health and the Environment, which through the ESE ISABU provides primary care services to the population that is unable to pay.

The ESE ISABU is composed of the following health centers: 24 Urban HC, 2 Mobile HC for the rural area, an external team in the urban area, an Intermediate Maternal and Child Health Care Center and a Local Hospital. The HC in the urban area are distributed throughout the city and located predominantly in areas with depressed socioeconomic conditions. Their management and administration are organized according to four regions: North, South, West and East. The primary activities of the HC are health promotion, specific protection, disease prevention, diagnostics and early treatment of low-complexity events. According to Colombia’s Mandatory Health Plan (POS, Spanish acronym) these correspond to primary level ambulatory care.

Health promotion and disease prevention programs for women’s sexual and reproductive health are priorities for health authorities in Bucaramanga. Since 2011, ESE ISABU has been investing significant financial and managerial efforts to adopt a PHC model (previously called “ISABU in your Neighborhood” and now called “Bucaramanga Grows with You”) to improve access, coordination, continuity and the comprehensiveness of care. In Bucaramanga, the PHC activities are carried out by the HC, whose priority programs are aimed at the maternal and child population.

The HC provide low-risk ANC, through which expectant mothers enter the system. If they are classified as low-risk they continue to receive care at the HC until 36 weeks of gestation, otherwise they are referred to the intermediate Maternal and Child Center. Through the EDCC program, women who need cytology are identified and admitted to the program, cytology is performed and the results are delivered. The program makes referrals in suspected cervical cancer cases. Through the FP program, users who can benefit from this program enter the system. It provides counseling and follow-up, in addition to supplying the family planning method to be used. The HC have a primary health care team (composed mostly of professionals in medicine, dentistry, nursing, nutrition and psychology, as well as nurse technicians) but do not have their own management teams. All of the management strategies are conducted by the central ESE ISABU administration, whose regional coordinators manage the resources and processes.

### Analysis of Technical Efficiency

The Data Envelopment Analysis (DEA) was used to analyze technical efficiency. This is a non-parametric technique to compare similar health centers based on information related to multiple inputs used to generate multiple products. The technical efficiency of the centers is calculated by dividing the weighted sum of the products by the weighted sum of the inputs. Since this is a non-parametric technique, the production function does not require a mathematical specification. The boundary of production possibilities, called the efficiency frontier, is determined from the combination of inputs and products pertaining to the centers with the best performance [19, 28]. The productivity of each center is calculated based on its distance from this frontier (the maximum level of efficiency observed in the sample). The centers located on the frontier, or those which determine it, are assigned an efficiency index of 1 (100 %) while indices lower than 1 (0-99 %) are assigned to centers below the frontier [11, 19, 28].

#### Variables in the DEA Model

The following variables were defined as inputs and outputs according to the conditions under which the ESE ISABU health centers operate and the products expected from the production processes used in the women’s prevention programs. (Additional file 1)

##### Inputs

Three variables were evaluated which correspond to the three main categories included in the analysis of DEA inputs: staff, capital and recurring or consumable goods [11]. These variables were measured in monetary terms such as the amount of resources the HC invest in the three input categories in order to produce the services for each prevention program. These three variables correspond to an aggregated measurement of a group of disaggregated variables, which are described in detail in the data collection section.

##### Outputs

Two variables were included— an indicator of production of services and a quality indicator. The production variable corresponds to the type of output most commonly used in the DEA analysis of health services [11]. The number of consultations or prevention activities served as the indicator of the production of services for each program evaluated. This included number of consultations provided by the ANC program, number of cervical-uterine cytologies performed by the EDCC program and number of counseling sessions conducted by the FP program (in medicine and nursing). A quality indicator was added to each model for two reasons. First, in order to compensate for analyses that only use variables related to the production of services or activities, which could determine that centers with a high production of activities are efficient when their quality performance is low. The second reason for including the quality measurement is the difficulty in measuring the ideal final outcome of the offering of health services— that is, the impact on the health of the population of interest. Therefore, the consensus in the literature is to recommend the use of an intermediate outcome measurement, such as quality of care indicators, which are known to be directly related to improving health [11, 29]. For the ANC program, the quality indicator was the percentage of adherence by antenatal care services to national ANC guidelines (Resolution 412 in the year 2000). The quality indicator for the EDCC program was the proportion of women who had at least one cytology performed over the previous three years. The indicator for the FP program was the proportion of women between 15 and 44 years of age who received at least one family planning counseling session at a health center. The detailed description of the measurement of the quality indicators for each health center is included in the data collection section.

#### Specifications of the DEA Model

A DEA model with variable returns-to-scale (VRS) was used. This assumes that a constant linear relationship does not exist between the inputs and outputs in the production process involved in the services offered by the three programs evaluated. This technical supposition was chosen because in the evaluation of technical efficiency by the present work: 1) the sizes of the HC evaluated varied greatly; 2) the management of the centers differed, which could interfere in the production process; and 3) the inclusion of a quality variable in the inputs is not consistent with a constant return to scale [11].

The efficiency analysis was focused on inputs because the decision-makers at the HC have more control over inputs than outputs. Thus, the analysis was aimed at describing to what degree the HC can reduce their inputs given the amount of outputs they provide in order to reach the production efficiency attained by other similar HC. Using this type of analysis, the DEA model calculates input “slacks” which refers to the excess inputs that each center could reduce in order to reach the efficiency frontier. The DEA model also calculates the number of times a center serves as a reference for peer centers. In addition to the efficiency score, an efficiency ranking was determined for the centers based on this information.

The analysis included a weighting limit of 20 % in the quality output defined for each program evaluated. This would prevent determining high efficiency scores for centers with a high production of activities (i.e. consultations) when the quality of their performance is low [30].

A separate DEA model was constructed for each prevention program. Banxia Frontier Analysis software [http://www.banxia.com/frontier/] was used to calculate the relative technical efficiency scores, inputs “slacks” and number of times serving a reference for each HC in each of the three models.

#### Efficiency Adjusted for Context Variables: A Four-Step Analysis

A relative efficiency analysis was performed and adjusted for the effect of context variables, or exogenous variables. This step was taken because these variables have been reported to have a direct influence on the performance of health centers. They affect production processes and lead to biased relative efficiency scores if not included in the analysis [27, 30, 31]. Using the Fried methodology [31], a four-step analysis was performed to calculate efficiency scores by adjusting for the context conditions at the HC.

We conducted a literature review for selecting the context variables that have been consistently associated with use of the three preventive programs: ANC [32–37], EDCC [38–42], and FP [43–49]. Based on the literature review, three contextual variables were commonly identified for the three programs: women’s age, educational level, and income/poverty level; for the FP program, having a stable union was also identified as an important contextual factor. We used the following variables as indicators of these contextual factors: women’s average age, proportion of women 18 or older with high school degree, proportion of population in Unidos program (the Colombian national program to fight poverty which support families with very low incomes), and the proportion of families with women as family head. The data collection section describes the sources and measurements of these variables. The four-step analysis was conducted as described below.

**Fig. 1:**
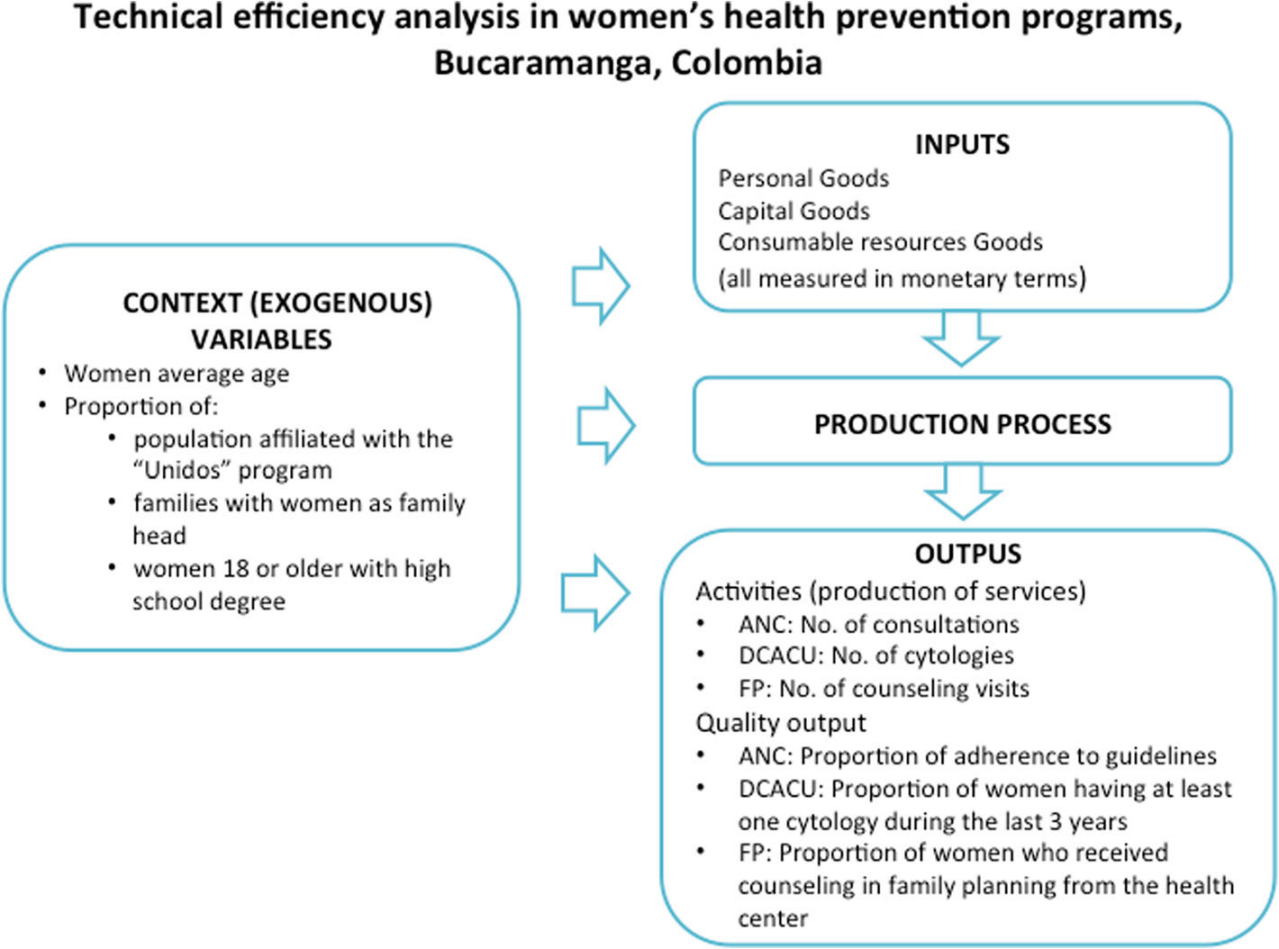
Components and variables for technical efficiency analysis of women´s health prevention programs in Bucaramanga, Colombia

Step 1. A DEA model was run for each prevention program with the input/output variables and technical specifications described in Sections 2.1.1 and 2.1.2, and the total input slacks were obtained from these models.
Step 2. The total input slacks obtained in Step 1 were used as dependent variables in a Tobit regression model, with the context variables as the independent variables. Tobit is a censored regression model designed to estimate linear relationships when the dependent variable is censored either above or below a threshold value; in this case the input slacks are censored above zero [50]. A separate Tobit regression was performed for each prevention program.
Step 3. The predicted slacks were calculated for each HC based on coefficients estimated by the Tobit model for each context variable. These estimations of the input slacks identify the total variation in the technical inefficiency measurements attributable to factors beyond the control of the centers’ management [31]. The difference between the maximum value of the predicted slack and the predicted slack was calculated for each HC and input type, and this difference was added to the original input values. The new adjusted values of the inputs corresponding to Staff, Capital and Goods (recurring or consumable) represent the inputs adjusted for the effects of the context variables [30, 31].
Step 4. A new DEA model was run using the adjusted inputs with the same outputs and technical specifications used in Step 1. These new measurements of the efficiency of the health centers include not only the adjustment for the quality of the activities but also for the influence of the variables external to the prevention programs’ production processes. The relationship among variables at each step is presented in Fig. 1.

#### Evaluation of the Robustness of the Efficiency Scores

A jackknife analysis, a resampling method for estimating the precision of a sample statistic, was used to verify the robustness of the efficiency scores obtained with the model. This was specified according to the potential effect of centers that behave as “extreme outliers”, which can affect the measurement of the efficiency scores [11, 51]. This was accomplished by removing one health center from the analysis each time and running a new DEA model, thereby obtaining new efficiency scores. The mean and standard deviations were calculated for the 21 models to measure the robustness of the final model.

### Data Collection

The data for all the HC input and output variables corresponded to the year 2012. The input variables for the three programs offered by each HC were collected from the institutions’ records on finances, employment, human resources, administration and quality. Information was obtained about financial expenses incurred by the ESE ISABU for each HC and about the staff, capital and recurring goods representing the inputs of each program (Colombian peso-COP, where one dollar equaled $1.797,79 COP in 2012). For the ANC program, the staff expenses included doctors, nurses, psychologists, nutritionists and dentists. The FP program included only medical and nursing staff and the EDCC program included only nursing. This accounts for the professionals responsible for developing each program. The number of staff in each profession was determined as well as the hours per profession and staff expenses incurred by the ISABU for each profession at each HC. This cost was calculated on a prorated basis for the year 2012. The percentage of consultations conducted by the programs in question was estimated based on the total number scheduled, and that percentage was applied to the total number of scheduled hours for the professionals at each HC. The costs related to capital goods were based on the value of the inventory as of December 31, 2012. This was calculated according to the percentage of scheduled consultations for the program. To this end, the total number of consultations was reviewed for each HC for the year 2012. Of the total scheduled consultations, the percentage conducted was estimated for the ANC program and that percentage was applied to the value of the total inventory for each HC as of December 31, 2012. The cost of recurring goods was determined based on the annual costs incurred by each HC in office supplies and inputs for each program (micronutrients for ANC, materials used in cytology for EDCC and birth control for FP).

To measure the outputs that represent indicators of production or activities, the physical and electronic health care records for the three programs at each HC were reviewed. Quantifications included the number of consultations offered by the ANC program, the number of cytologies performed by the EDCC and the total number of family planning counseling sessions conducted by the FP program (medicine and nursing).

To measure the outputs that represent indicators of the quality of the programs, a sample of prenatal clinical histories from 2012 was reviewed for the ANC program. For the EDCC and FP programs, interviews were conducted of a sample of the users of these programs. For the ANC program, a random sample of 16 to 60 histories was selected, depending on the size of the HC, resulting in the review of a total of 822 histories (out of a total of 2932 pregnant women who received care in 2012). The prenatal histories were reviewed by the medical and nursing professionals who participated in creating a checklist based on the National ANC protocol guidelines (Resolution 412 from the year 2012). The percentage of adherence to this norm was then determined for each one. For the ANC program, the quality output for each HC was calculated based on the average percentage of adherence by antenatal care to the national guidelines. For the EDCC and FP programs, users of each HC between the ages of 15 and 65 were randomly selected, whose first contact with the HC during 2012 was a consultation with a doctor (excluding users of antenatal care services). A total of 872 women were contacted, of which 558 were eligible to respond to the FP questionnaire and 740 to the EDCC questionnaire. For the EDCC program, the proportion was calculated of women interviewed from each HC who were eligible for a cytology and who reported having undergone at least one during the previous three years. This indicator was selected since it corresponds to the EDCC program’s national coverage goal (Resolution 412). For the FP program, the proportion was calculated of women interviewed from each HC between 15 and 45 years of age who had received at least one FP counseling session from the health centers.

The context variables for the HC were based on the information collected at the beginning of the year 2013 by the “Buacaramanga Grows with You” strategy, which is the implementation strategy for the primary health care model in the municipality of Bucaramanga. This strategy conducted a door-to-door survey in the city’s neighborhoods in order to characterize the families and persons who were enrolled in the programs offered by the ESE ISABU’s HC. The electronic database from this municipal strategy was used to obtain the basic information for calculating the four context variables for each HC: average age of the women, proportion of population affiliated with the “Unidos” program to fight poverty, proportion of families whose head of household is female, and proportion of women 18 years or older who completed secondary school.

## Results

This study analyzed the technical efficiency of 21 HC belonging to the ESE ISABU in the city of Bucaramanga. These are primary care centers which offered activities related to prevention programs for women during the year 2012. Table 1 presents a description of the variables included in the analysis. Table 2 presents the output/input ratio and the distribution of the technical efficiency scores for each prevention program at each HC, only adjusted for a 20 % minimum weighting of the quality variable (baseline score). This baseline analysis corresponds to Step 1 in the Fried method. The average efficiency score (SD = standard deviation) was 95.8 % (SD = 0.095) for the ANC program, 99.6 % (SD = 0.010) for the EDCC program and 90.9 % (SD = 0.159) for the FP program.

**Table 1:** Descriptive statistic of input, output and context variables, Bucaramanga, Colombia, 2012

Variable	Mean	Std. Deviation	Minimum	Maximum
Inputs				
Personnel (COP)				
ANC Program	15987421.30	12412112.36	3124380.18	54555970.55
DCACU Program	7919344.57	4808720.61	1741427.31	21405561.67
FP Program	11211856.54	4848131.55	1835309.87	23996440.01
Capital (COP)				
ANC Program	4522934.74	3810770.69	802426.48	17205338.44
DCACU Program	3306247.94	2101757.80	1460307.53	10835288.96
FP Program	12309640.44	11129064.97	3124374.21	40527398.70
Consumable resources (COP)				
ANC Program	270265.14	212771.99	47526.00	728285.00
DCACU Program	1104567.53	612587.02	325973.48	2670384.17
FP Program	15465950.78	8640885.96	3501547.00	35408461.20
Outputs				
Activities output				
ANC: No. of consultations	740.57	585.86	98.00	2081.00
DCACU: No. of citologies	648.57	367.39	177.00	1582.00
FP: No. of counseling visits	1226.09	590.33	168.00	2516.00
Quality output				
ANC: Proportion of adherence to guidelines	0.75	0.04	0.63	0.81
DCACU:Proportion women with 3:1 squeme^a^	0.74	0.14	0.29	0.88
FP: Proportion counseling coverage^b^	0.77	0.13	0.52	1.00
Context variables				
Women average age	34.39	3.04	29.90	40.30
Proportion of population in Unidos program^c^	0.08	0.08	0.00	0.23
Proportion of families with women as family head	0.63	0.07	0.43	0.75
Proportion of women 18 or older with high school degree	0.48	0.04	0.44	0.60

*ANC* antenatal care, *DCACU* cancer of the uterine cervix diagnosis, *FP* Family Planning

^a^ squeme 3:1 refers to women having at least one citology during the last 3 years

^b^ Proportion of women who received counseling in family planning from the health center

^c^Unidos program is the Colombian national program to fight poverty which support families with very low incomes

**Table 2:** Technical efficiency scores, reference ranking and inputs slacks corresponding to the baseline analysis, by health center and program, Bucaramanga, Colombia. 2012

Health center	ANC Program						DCACU Program						FP Program					
	0	1	2	3	4	5	0	1	2	3	4	5	0	1	2	3	4	5
	Output/Input ratio^a^	DEA Score	Ref N^b^	Personal Slacks	Capital Slacks	Recurrent expenses Slacks	Output/ Input ratio^a^	DEA Score	Ref N^b^	Personal Slacks	Capital Slacks	Recurrent expenses Slacks	Outputs/Inputs ratio^a^	DEA Score	Ref N^b^	Personal Slacks	Capital Slacks	Recurrent expenses Slacks
Antonia Santos	21.69	0.888	0	−11	−73	−11	36.45	1	4	0	0	0	18.07	1	2	0	0	0
Bucaramanga	40.34	1	1	0	0	0	91.96	1	2	0	0	0	33.42	1	3	0	0	0
Campo Hermoso	48.70	1	7	0	0	0	74.88	1	6	0	0	0	26.09	0.667	0	−33	−33	−33
Colorados	42.63	0.959	0	−4	−4	−4	117.84	0.987	0	−1	−1	−6	51.64	1	2	0	0	0
Comuneros	46.70	1	1	0	0	0	116.03	1	4	0	0	0	44.20	1	2	0	0	0
Concordia	23.53	1	2	0	0	0	130.08	1	1	0	0	0	53.07	1	6	0	0	0
El Rosario	54.71	1	3	0	0	0	19.54	1	1	0	0	0	14.74	0.661	0	−33	−80	−33
Gaitan	45.32	1	2	0	0	0	133.20	0.997	0	−34	0	0	58.77	1	3	0	0	0
Girardot	37.04	0.970	0	−2	−2	−2	52.58	1	1	0	0	0	29.79	0.713	0	−28	−83	−28
IPC	51.74	1	4	0	0	0	81.85	1	1	0	0	0	45.63	1	1	0	0	0
Kennedy	32.45	1	5	0	0	0	18.21	1	1	0	0	0	9.07	1	4	0	0	0
La Joya	18.61	1	2	0	0	0	19.92	0.999	0	−58	0	0	25.71	0.991	0	0	0	0
La Libertad	13.54	1	1	0	0	0	66.22	1	1	0	0	0	18.66	1	3	0	0	0
Morrorico	30.20	0.948	0	−5	−5	−5	96.72	0.981	0	−24	−1	−1	47.41	1	1	0	0	0
Mutis	42.61	0.874	0	−12	−12	−47	19.86	1	1	0	0	0	23.76	1	2	0	0	0
Pablo VI	21.53	1	2	0	0	0	47.51	1	1	0	0	0	26.43	1	1	0	0	0
Regaderos	38.23	1	3	0	0	0	83.41	1	1	0	0	0	47.20	1	5	0	0	0
San Rafael	27.32	1	3	0	0	0	18.62	1	5	0	0	0	47.37	1	6	0	0	0
Santander	24.37	1	1	0	0	0	135.89	1	3	0	0	0	21.36	0.755	0	−24	−24	−24
Toledo Plata	20.31	0.583	0	−58	−58	−41	14.80	1	1	0	0	0	12.55	0.462	0	−53	−63	−53
Villa Rosa	31.26	0.892	0	−16	−10	−10	57.36	0.957	0	−45	−4	−4	34.70	0.843	0	−15	−15	−15

^a^Output/Input ratio: (activity output / sum of personal, capital, recurrent expenses inputs )*1000000

^b^Number of times being health center’s frontier referent at baseline analysis

In the second step of the analysis, which was to adjust for context, the Tobit regression estimated the effect of the contextual variables on the input slacks resulting from the baseline DEA. Table 3 presents the coefficients estimated and their corresponding 95 % confidence intervals (95 % CI) for each program. In general, the positive coefficients indicate increasing inefficiency (more slacks) as the values of the variables increase, while negative coefficients indicate increasing efficiency (fewer slacks) as the values of the variables increase. Thus, the results for the ANC program show that the educational level and women’s age affect more negatively the efficiency of the centers (valid for the three inputs included in the study). For the EDCC program, the women’s age has a more negative effect on the technical efficiency of the HC. For the FP program, educational level was related to higher levels of efficiency at the HC, and was notable for all three types of inputs. The McFadden’s R^2^ for all models ranged between 0.02 and 0.07, except for the models for Capital slacks (R^2^ = 0.20) and Recurring goods slacks (R^2^ = 0.24) for the EDCC program. Nevertheless, none of the context variables presented a statistically significant coefficient in relation to the technical efficiency of the HC.

**Table 3:** Tobit regression analysis of potential contextual factors influencing the input slacks produced by the baseline DEA model

	ANC Program									EDCC Program									FP Program								
	Staff Slacks			Capital Slacks			Recurring Goods Slacks			Staff Slacks			Capital Slacks			Recurring Goods Slacks			Staff Slacks			Capital Slacks			Recurring Goods Slacks		
Context variable	Coef	95 % CI		Coef	95 % CI		Coef	95 % CI		Coef	95 % CI		Coef	95 % CI		Coef	95 % CI		Coef	95 % CI		Coef	95 % CI		Coef	95 % CI	
Age^a^	1.85	−10.2	13.9	3.50	−14.9	21.9	1.39	−10.9	13.7	0.73	−18.8	20.26	0.50	−3.02	4.02	1.24	−4.46	6.94	4.49	−8.57	17.55	9.25	−10.4	28.9	4.49	−8.57	17.55
Education^b^	280.5	−342.8	903.9	633.9	−331.8	1599.	389.9	−265	1045	−399.6	−1455	656.4	−22.8	−143	97.5	−1.48	−174	171	−331.1	−1070	408.6	−580	−1683	523	−331	−1070	408
Poverty^c^	−4.48	−426.9	417.9	77.72	−565.3	720.7	25.65	−413	464	−282.7	−1077	512.2	−33.2	−116	49.94	−33.9	−146	78.5	101.5	−431.4	634.6	204	−586	995	101.5	−431	634
Stable Union^d^										−197.1	−606.9	212.7	−10.3	−49.2	28.56	2.24	−53.5	57.9									
Constant	−182	−781	415	−409	−1281	461	−222	−800	355	349.9	−808	1508	8.22	−150	166	−33	−278	212	16.7	−735	768	−26.45	−1159	1106	16.76	−735	768

^a^Age = Women’s average age

^b^Education = Proportion of women 18 or older with high school degree

^c^Poverty = Proportion of population in Unidos program, the Colombian national program to fight poverty which support families with very low incomes

^d^Stable union = Proportion of families with women as family head

The predicted slacks resulting from the Tobit regressions for each input and program were used to obtain new adjusted input values, which resulted in an increase between 4 % and 120 % for staff, 4 % and 22 % for capital and 1 % and 22 % for the recurring goods for the three programs.

Table 4 presents the technical efficiency scores from the DEA models adjusted by weighting the quality variable and for the effect of the context input variables. The resulting average adjusted efficiency score (SD) was 92.4 % (SD = 0.121) for the ANC program, 97.5 % (SD = 0.045) for the EDCC program and 86.2 % (SD = 0.198) for the FP program. On each program, 12 of the 21 HC (57.1 %) were 100 % efficient. For the ANC program, 3 of the 9 HC that did not reach 100 % efficiency had an efficiency score under 75 %. For the EDCC program, only 1 of 9 HC that did not reach 100 % efficiency had a score under 90 %, and for the FP program 6 of 9 HC that did not reach 100 % efficiency had scores under 75 %. In terms of the ranking of centers by the number of times they acted as a reference in the models, the most frequent centers were El Rosario y Campo Hermoso for the ANC program, Comuneros and La Libertad for the EDCC program and San Rafael, Concordia, and La Libertad for the FP program (Table 4).

**Table 4:** Adjusted technical efficiency scores and reference rankings, by health center and program, Bucaramanga, Colombia, 2012

Health center	ANC Program		EDCC Program		FP Program	
	1	2	1	2	1	2
	Adjusted Score	Reference number	Adjusted Score	Reference number	Adjusted Score	Reference number
Antonia Santos	0.733	0	0.990	0	1.000	2
Bucaramanga	0.865	0	1.000	1	0.904	0
Campo Hermoso	1.000	7	1.000	3	0.587	0
Colorados	1.000	1	0.983	0	1.000	1
Comuneros	1.000	1	1.000	8	1.000	5
Concordia	1.000	6	1.000	3	1.000	7
El Rosario	1.000	8	1.000	1	0.396	0
Gaitan	0.846	0	0.935	0	1.000	4
Girardot	0.943	0	1.000	2	0.630	0
IPC	1.000	2	0.961	0	1.000	1
Kennedy	1.000	4	1.000	1	1.000	4
La Joya	1.000	6	0.968	0	0.738	0
La Libertad	1.000	2	1.000	7	1.000	7
Morrorico	0.980	0	0.907	0	1.000	2
Mutis	0.652	0	1.000	3	1.000	3
Pablo VI	0.955	0	1.000	1	0.973	0
Regaderos	1.000	3	1.000	1	1.000	4
San Rafael	1.000	3	0.911	0	1.000	9
Santander	1.000	4	1.000	6	0.776	0
Toledo Plata	0.519	0	0.942	0	0.424	0
Villa Rosa	0.926	0	0.876	0	0.668	0

The jackknife analysis presented robust technical efficiency results with the adjusted DEA model, having showed very little variations when omitting any of the HC from the analysis (Table 5).

**Table 5:** Mean scores of technical efficiency from Jackknife analysis, by health center and program

Health center	ANC Program		EDCC Program		FP Program	
	Mean	SD	Mean	SD	Mean	SD
Antonia Santos	0.738	0.015	0.991	0.003	1.000	0.000
Bucaramanga	0.879	0.040	1.000	0.000	0.914	0.029
Campo Hermoso	1.000	0.000	1.000	0.000	0.588	0.003
Colorados	1.000	0.000	0.985	0.005	1.000	0.000
Comuneros	1.000	0.000	1.000	0.000	1.000	0.000
Concordia	1.000	0.000	1.000	0.000	1.000	0.000
El Rosario	1.000	0.000	1.000	0.000	0.397	0.005
Gaitan	0.862	0.045	0.937	0.008	1.000	0.000
Girardot	0.949	0.017	1.000	0.000	0.648	0.081
IPC	1.000	0.000	0.965	0.012	1.000	0.000
Kennedy	1.000	0.000	1.000	0.000	1.000	0.000
La Joya	1.000	0.000	0.970	0.007	0.745	0.025
La Libertad	1.000	0.000	1.000	0.000	1.000	0.000
Morrorico	0.983	0.006	0.909	0.009	1.000	0.000
Mutis	0.672	0.077	1.000	0.000	1.000	0.000
Pablo VI	0.960	0.013	1.000	0.000	0.978	0.011
Regaderos	1.000	0.000	1.000	0.000	1.000	0.000
San Rafael	1.000	0.000	0.913	0.008	1.000	0.000
Santander	1.000	0.000	1.000	0.000	0.787	0.049
Toledo Plata	0.523	0.012	0.943	0.003	0.427	0.009
Villa Rosa	0.932	0.017	0.877	0.005	0.674	0.015

## Discussion

The present study made it possible to evaluate the technical efficiency of the performance of the women’s health prevention programs offered by ESE ISABU health centers during the year 2012.

Our findings demonstrate a large degree of heterogeneity in the performance levels of the health centers included in the study. At 5 HC (24 %), all three programs consistently had relative technical efficiency levels of 100 % (3 of these HC were located in the most depressed areas of the city); at 8 HC (38 %) 2 programs were efficient; at another 5 HC (24 %) at least one program was efficient; and at 3 HC (14 %) none of the programs attained efficient levels. In general, the EDCC program had the highest scores and FP had the lowest. Nevertheless, our findings cannot be compared with other studies since, to our knowledge, this is the first study to apply a DEA efficiency analysis to women’s health care programs aimed at health promotion and disease prevention at the primary care level. The efficiency scores obtained are higher than other investigations performed at primary care centers in the Americas that included some ANC and FP outcome indicators [18, 19, 24].

The findings by the present study show the effect of adjusting efficiency estimations for quality variables and for the context variables at the health centers in the analysis. Similar to previous studies, our results affirm the need to make these adjustments in order to prevent biased estimations [27, 30, 52, 53]. The coefficients resulting from the Tobit models were not statistically significant probably as a consequence of the small sample of centers evaluated; in addition the McFadden’s R^2^ were very small for most of the models probably because as a pseudo-R^2^ measure this statistic is usually lower and should not be interpreted in the same way as the R^2^ from an ordinary least square regression. The results, however, suggest that the adjustment for context variables derived from the Tobit models have an important effect given that they modified the efficiency scores and rankings of the centers.

The direction of the coefficients obtained with the Tobit models enabled us to determine the degree to which the context variables affected the (in)efficiency of each of the programs offered by each HC [27, 31]. For the EDCC program, our results found that increased average of women’s age was associated with higher levels of inefficiency at the HC. For the FP program, having high school degree was associated with higher levels of efficiency at the HC. And for ANC, older age and having high school degree negatively affected the centers’ efficiency outcomes. Our results coincide with other studies, which also demonstrate that including context variables dramatically changes the conclusions about the performance of health centers and, therefore, ignoring or not considering these factors can lead to incorrect performance measurements [27, 30, 52, 53].

These results offer valuable information for decision-makers by providing evidence of a suboptimal utilization of resources at some centers, which may be related to organizational as well as management factors within the ESE ISABU and/or at the centers themselves, such as more structural characteristics or determinants. Therefore, in terms of the implications for policies, our results suggest that health managers need to take into account contextual factors when designing strategies to strengthen the prevailing PHC model, in order to ensure the optimal allocation of resources to the health centers. This will maximize their impact on the health of the population and, in particular, improve local health services for women. Conditions related to the persistence of poverty and poor access to education and health insurance need to be changed, among others, while management mechanisms within the institutions need to be strengthened.

Some of the strengths of this study are worth highlighting, such as the inclusion of quality variables that are not routinely measured by the HC. For example, for ANC, the averages of the programs’ adherence to health care protocol were calculated; and for the EDCC and FP programs, coverage was calculated based on a sampling of women who had visited the HC for a medical consultation in 2012 and who were eligible for these programs. Furthermore, most studies about the technical efficiency of primary care centers only use workforce variables as input variables. In addition to these, our study also included inputs used by providers to improve health, such as the monetary value of infrastructure and recurring goods and services, micronutrients supplied, lab tests conducted and birth control methods provided, among others.

Another strength of this investigation was the inclusion of context and quality variables, which enabled measuring their effects on the (in)efficiency of the implementation of the women’s health programs at the HC. Including these variables resulted in more robust efficiency scores. In addition, the application of the jackknife analysis to the efficiency scores obtained strengthens the estimations generated, as described previously in the literature [11, 51].

Nevertheless, our study is not without its limitations. The main limitation is the small number of centers included in the analysis, which limit the statistical inferences derived from the Tobit models in Step 3 and therefore might introduce some additional random error in estimations in Step 4. In addition, because of the high number of efficient HC and therefore null slacks values, the Tobit regression analysis could not be adjusted for errors using the bootstrap method suggested by Cordero-Ferrera [30]. An alternative method to measure technical efficiency is the Stochastic Frontier Analysis (SFA), which is indicated when severe measurement error is present and might be separated from the technical efficiency measure, and there is an assumption of good fit to classical functional forms of production. DEA in turn, is preferred when measurement error has a low probability to occur and the assumptions of classical production are not met. Given that SFA controls for measurement error, DEA scores are usually higher than SFA scores [16]. Unfortunately, the small sample size did not allow us to conduct a sensitivity analysis using SFA. Despite this important limitation, results of this study using DEA analysis are probably a good estimation of the ISABU health center’s technical efficiency because introducing quality and contextual variables minimized measurement error and the use of quality outputs clearly violates the classical economical function of production.

Finally, because of the limitations in the information, some variables that may affect the performance of the HC could not be included in the analysis, such as the financial structure of the programs, methods used to determine the pay of health professionals and types of employment. These variables could play an important role in the estimations of efficiency given that the Colombian health model is oriented towards a free-market competition model.

## Conclusions

A large degree of heterogeneity was found in the performance of the women’s health programs offered by the HC belonging to the ESE ISABU. In general, better scores were observed for the EDCC program and a need was identified to strengthen the management and operations of the other programs, particularly the FP program which systematically had the lowest scores.

From the qualitative point of view (since statistically significant differences were not found), the context variables evaluated were found to affect the (in)efficiency scores of the HC. After adjusting for these variables, these scores were generally found to decrease and the ranking of the HC changed considerably. This finding supports reports by previous studies of the need to adjust for these variables as well as for variables that measure the quality of the performance of these programs.

This type of evidence is important to strengthening the management, organization, and planning processes involved in local PHC services, in the context of a market model such as that operating in Colombia.

## Additional file

Additional file 1: Input and output variables for DEA analysis in three women´s health programs. (XLS 47 kb)
